# X-ray Structures and Computational Studies of Two Bioactive 2-(Adamantane-1-carbonyl)-*N*-substituted Hydrazine-1-carbothioamides

**DOI:** 10.3390/molecules27238425

**Published:** 2022-12-01

**Authors:** Lamya H. Al-Wahaibi, Kowsalya Alagappan, Olivier Blacque, Ahmed A. B. Mohamed, Hanan M. Hassan, María Judith Percino, Ali A. El-Emam, Subbiah Thamotharan

**Affiliations:** 1 Department of Chemistry, College of Sciences, Princess Nourah Bint Abdulrahman University, Riyadh 11671, Saudi Arabia; 2 Biomolecular Crystallography Laboratory, Department of Bioinformatics, School of Chemical and Biotechnology, SASTRA Deemed University, Thanjavur 613 401, India; 3 Department of Chemistry, University of Zurich, Winterthurerstrasse 190, 8057 Zurich, Switzerland; 4 Department of Medicinal Chemistry, Faculty of Pharmacy, Mansoura University, Mansoura 35516, Egypt; 5 Department of Pharmacology and Biochemistry, Faculty of Pharmacy, Delta University for Science and Technology, International Costal Road, Gamasa City, Mansoura 11152, Egypt; 6 Unidad de Polímeros y Electrónica Orgánica, Instituto de Ciencias, Benemérita Universidad Autónoma de Puebla, Val3-Ecocampus Valsequillo, Independencia O2 Sur 50, San Pedro Zacachimalpa, Puebla 72960, Mexico

**Keywords:** adamantane, hydrazine-1-carbothioamide, Hirshfeld surface, CLP–Pixel, QTAIM, molecular docking, urease inhibition, antiproliferative agents, H-H bonding

## Abstract

Two biologically active adamantane-linked hydrazine-1-carbothioamide derivatives, namely 2-(adamantane-1-carbonyl)-*N*-(*tert*-butyl)hydrazine-1-carbothioamide) **1** and 2-(adamantane-1-carbonyl)-*N*-cyclohexylhydrazine-1-carbothioamide **2,** have been synthesized. X-ray analysis was conducted to study the effect of the *t*-butyl and cyclohexyl moieties on the intermolecular interactions and conformation of the molecules in the solid state. X-ray analysis reveals that compound **1** exhibits folded conformation, whereas compound **2** adopts extended conformation. The Hirshfeld surface analysis indicates that the contributions of the major intercontacts involved in the stabilization of the crystal structures do not change much as a result of the *t*-butyl and cyclohexyl moieties. However, the presence and absence of these contacts is revealed by the 2D-fingerprint plots. The CLP–Pixel method was used to identify the energetically significant molecular dimers. These dimers are stabilized by different types of intermolecular interactions such as N–H···S, N–H···O, C–H···S, C–H···O, H–H bonding and C–H···π interactions. The strength of these interactions was quantified by using the QTAIM approach. The results suggest that N–H···O interaction is found to be stronger among other interactions. The in vitro assay suggests that both compounds **1** and **2** exhibit urease inhibition potential, and these compounds also display moderate antiproliferative activities. Molecular docking analysis shows the key interaction between urease enzyme and title compounds.

## 1. Introduction

Hydrazine-1-carbothioamide (3-thiosemicarbazide) derivatives received considerable attention owing to their diverse chemotherapeutic activities [1]. Several mono- and di-substituted-*N*-hydrazine-1-carbothioamides were reported to possess marked anticancer [2,3,4,5,6,7,8,9], antifungal [10,11,12], antituberculous [13,14,15], antiviral [16,17], antibacterial [18,19,20,21,22], and antiprotozoan activities [23,24,25,26]. In addition, several hydrazine-1-carbothioamide derivatives were recognized as potent urease inhibitors [27,28,29,30,31,32].

On the other hand, numerous adamantane-based drugs are currently used as efficient chemotherapies for the control of various pathological disorders [33,34,35]. Amantadine [36] and rimantadine [37] were discovered early to be effective therapies against Influenza A viral infections and tromantadine as antiviral drug for the treatment of skin infection caused by herpes simplex virus [38]. In addition, the adamantane-based drugs adaphostin [39], adarotene [40], and opaganib [41] are currently used as efficient therapies against resistant cancers.

Urease (urea amidohydrolase) is a nickel ion-dependent enzyme that catalyzes the hydrolysis of urea to ammonia and carbamate [42]. Ureases are widespread in nature among plants, bacteria, fungi, algae, and invertebrates. In humans, excessive urease activity is associated with the development of numerous health problems including stomach cancer [43], peptic ulcers [44], hepatic encephalopathy, and hepatic coma [45].

Based on the abovementioned findings, two substituted hydrazine-1-carbothioamide derivatives carrying a highly lipophilic adamantane cage and bulky substituents (*t*-butyl and cyclohexyl) were prepared and their in vitro urease inhibitory and antiproliferative activities were evaluated. Furthermore, single-crystal X-ray diffraction (XRD) studies were performed for these compounds to determine the effect of the *t*-butyl and cyclohexyl substituents on the molecular conformation and intermolecular interactions. The substituted thiosemicarbazide derivatives with the formula R_1_–(C=O)–NH–NH–(C=S)–NH–R_2_ comprise the units of amide and thiourea that occupy the central part of the molecules. These moieties show a variety of hydrogen bonding interactions, including N–H···O=C (amide···amide and amide···thiourea) and N–H···S=C (amide···thiourea) bonds in addition to weak C–H···O=C and C–H···S=C interactions. A Cambridge Structural Database (CSD version 5.43, November 2021, update June 2022) [46] search was conducted by using the general formula R_1_–(C=O)–NH–NH–(C=S)–NH–R_2_, and the search yielded 106 hits. After excluding cocrystal and hydrate molecules, there were approximately 71 hits, which clearly indicated the scope for the generation of novel molecules with different R_1_ and R_2_ groups. It has been observed that the molecular conformation of these derivatives, especially the central moiety (amide and thiourea) exhibits folded and extended conformations in the solid state depending on the nature of substituent groups R_1_ and R_2_.

Ashokkumar et al. developed the BODIPY dye linked with a strong hydrogen bond donor amidothiourea receptor to selectively detect the fluoride anion compared to other anions such as AcO^−^ and H_2_PO_4_^−^ [47]. Basu et al. synthesized two amidothiourea-based colorimetric receptors for basic anions such as F^−^, AcO^−^, and H_2_PO_4_^−^ and described the deprotonation of the amide NH proton [48]. In this study, the authors used the substituent R_1_ being 3,5-dinitrophenyl and R_2_ was phenyl and naphthyl moieties. The deprotonation of amide NH was proven with the help of a salt crystal of amidothiourea with naphthyl and tetrabutylammonium. Recently, Pitucha et al. synthesised a series of thiosemicabazide derivatives and investigated their antitubercular activities [49]. The type II β-turn motif, an intramolecular N–H···O hydrogen bond was observed in glycinephenylalanine dipeptide-based *N*-amidothioureas [50]. In the present investigation, we describe the crystal structures of two molecules that possess urease inhibitory and antiproliferative activities. Various dimeric motifs were formed by different types of intermolecular interactions identified from the crystal structures by using the CLP–Pixel method. In addition, density functional theory (DFT) calculations and the quantum theory of atoms in molecules (QTAIM) approach were used to study the energetics and nature of interactions observed in these structures. Hirshfeld surface analysis was used primarily to delineate the effects of the *t*-butyl and cyclohexyl moieties on the intermolecular interactions in the solid state.

## 2. Results and Discussion

### 2.1. Chemical Synthesis

**Scheme 1:**
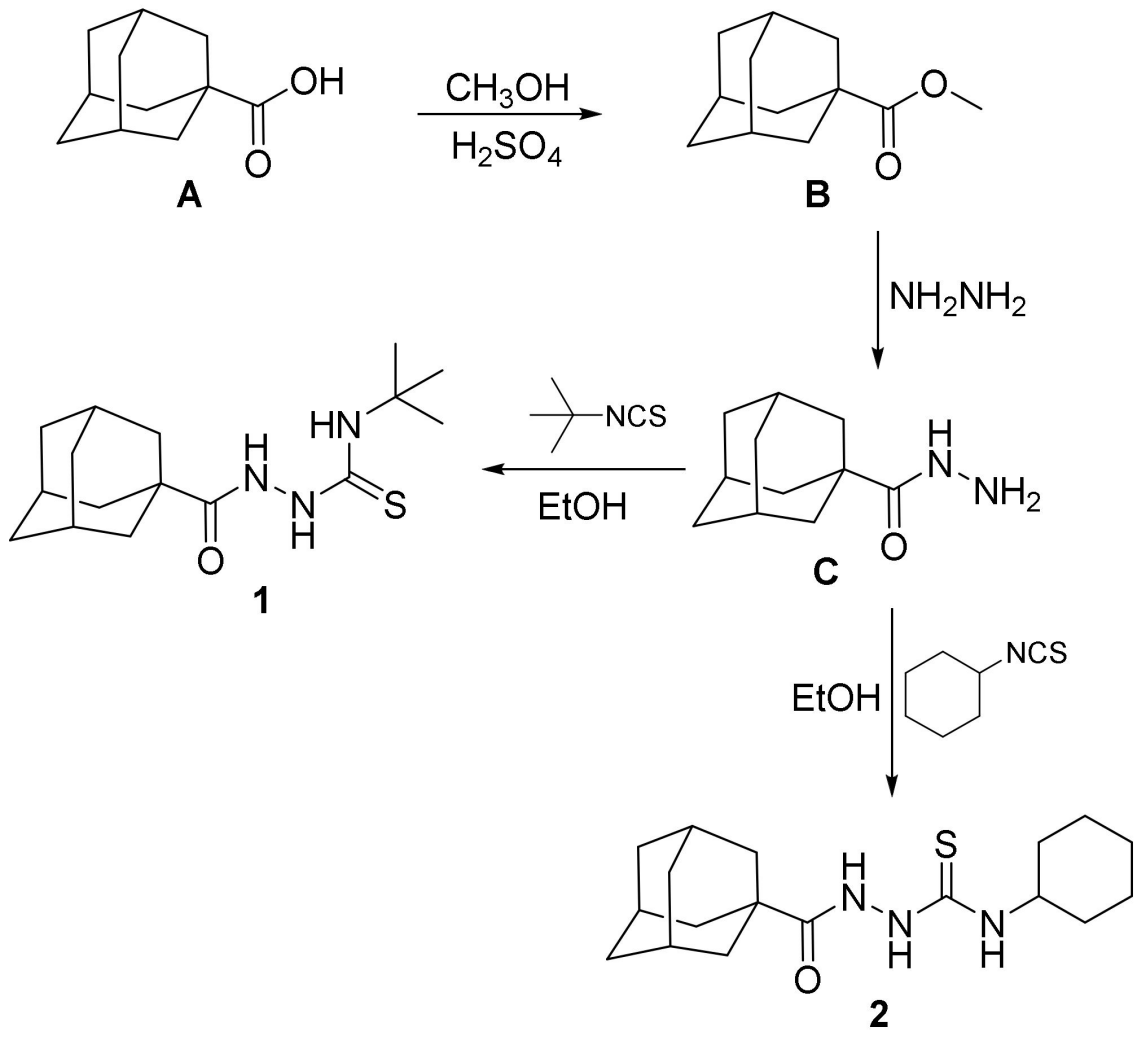
The synthetic pathway for the investigated compounds **1** and **2**.

The investigated compounds **1** and **2** were prepared staring with adamantane-1-carboxylic acid **A** via esterification with methanol in the presence of sulfuric acid to yield the methyl ester **B**. The methyl ester **B** was then reacted with hydrazine hydrate to yield adamantane-1-carbohydrazide **C [51,52]**. Adamantane-1-carbohydrazide **C** was then reacted with equimolar amounts of *t*-butyl isothiocyanate or cyclohexyl isothiocyanate via heating in ethanol to yield the target compounds **1** and **2** (Scheme 1).

### 2.2. Molecular and Crystal Structures

**Figure 1:**
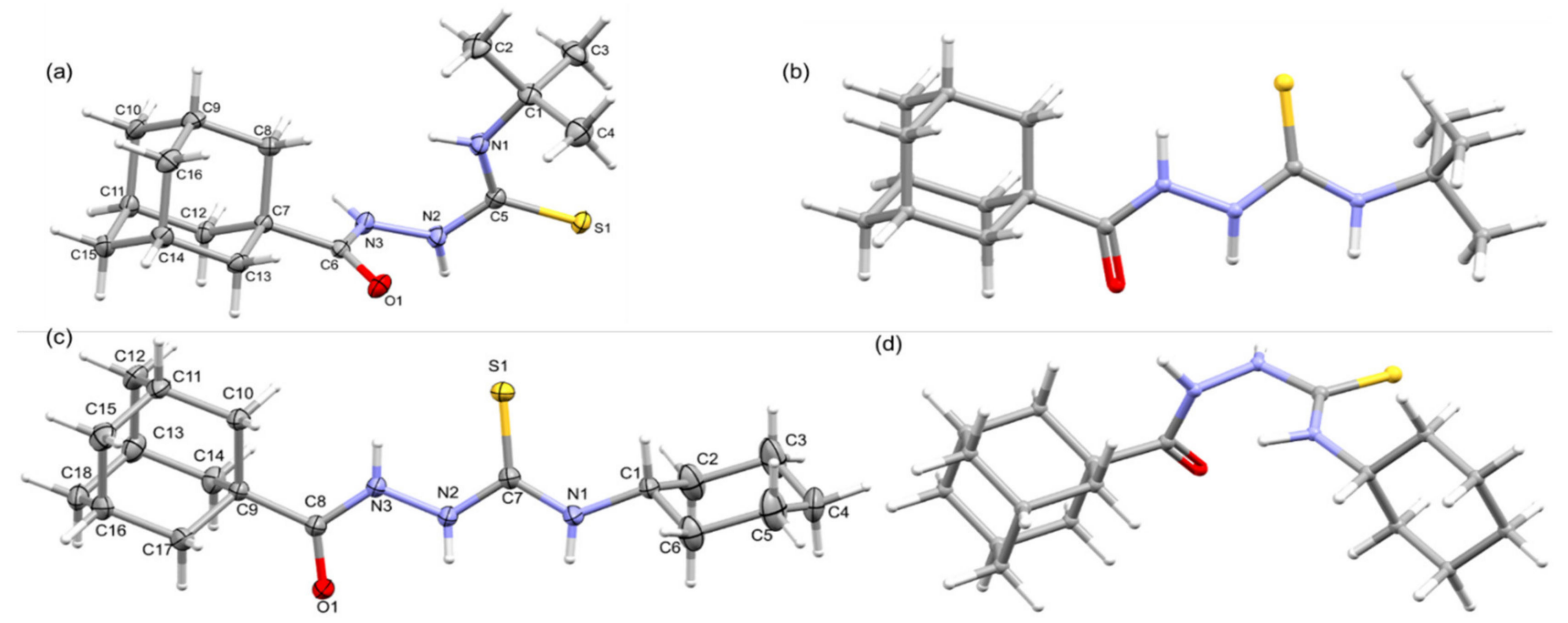
Thermal ellipsoid plot of (**a**) compound **1** and (**c**) compound **2**, drawn with 50% probability level, (**b**) the optimized structure of **1** in conformation of **2** (conformer 1a), and (**d**) the optimized structure of **2** in conformation of **1** (conformer 2a).

The crystal structures of these compounds **1** and **2** were determined at low temperatures (160 K). The X-ray crystallographic analysis reveals that both compounds crystallize in the triclinic system. Crystal data and refinement parameters for these compounds are summarized in Table 1 and the ORTEP representations are illustrated in Figure 1. Compounds **1** and **2** differ by the substituents namely *tert*-butyl and cyclohexyl, respectively. The X-ray structures reveal conformational differences around the thioamide moiety, resulting in folded conformation for compound **1** and an extended conformation for compound **2** as shown in Figure 1a,c.

#### 2.2.1. Molecular Conformations

More specifically, the amide (O1=C6–N3–H3 = 177.13°), hydrazine (H3–N3–N2–H2 = −89.72°), and thiourea (H2–N2–C5–S1 = 2.13° and S1=C5–N1–H1 = 174.38°) groups adopt trans, clinal, and syn/anti conformations, respectively, in compound **1**, whereas the corresponding groups (amide: O1=C8–N3–H3 = −178.33°; hydrazine: H3–N3–N2–H2 = 168.01°; thiourea: H2–N2–C7=S1 = −170.92° and S1=C7–N1–H1 = 178.53°) adopt a trans conformation in **2**. The variation in the molecular conformation could also affect the bond lengths of the central unit, which bridge adamantane and the cyclohexyl/*tert*-butyl groups. The selected bond lengths involving the central unit is summarized in Table 2. This table shows that out of eight bonds, bond lengths of six bonds are shortened in **2** compared to structure **1**. We note that the C8–N3 bond is significantly reduced in **2**, and the N3 atom is part of amide and hydrazine units. The latter group exhibits a clinal conformation rather than a trans conformation.

We carried out computational modelling to understand the conformational stability and find the energy difference between two different conformations, i.e., folded and extended conformations. First, we performed single-point energy calculations for X-ray structures of **1** (conformer 1) and **2** (conformer 2) with M062X-D3/def2-TZVP level of theory. Then we replaced the *tert*-butyl group with cyclohexane in **1**, and the resultant model (conformer 2a) was fully optimized with the same level of approximation. Similarly, we substituted the cyclohexyl group with the *tert*-butyl group in **2**, and the resulting model (conformer 1a) was freely optimized in the gas phase. Positive vibrational frequencies indicate that the optimized models are the conformers with the lowest potential energy. The results suggest that when cyclohexyl is present in the structure, the structure exhibits a folded conformation that is relatively more stable than extended conformation. The relative energy difference between the folded and extended conformations is about 2.1 kcal mol^−1^. In contrast, when *tert*-butyl is present, the extended conformation is relatively more stable than the folded conformation. The energy difference between these two conformers is 3.3 kcal mol^−1^. The electronic structures also display bond length variations for bonds of the central core region due to conformational differences, as noted in X-ray structures.

#### 2.2.2. Hirshfeld Surface and 2D-Fingerprint Plots

**Figure 2:**
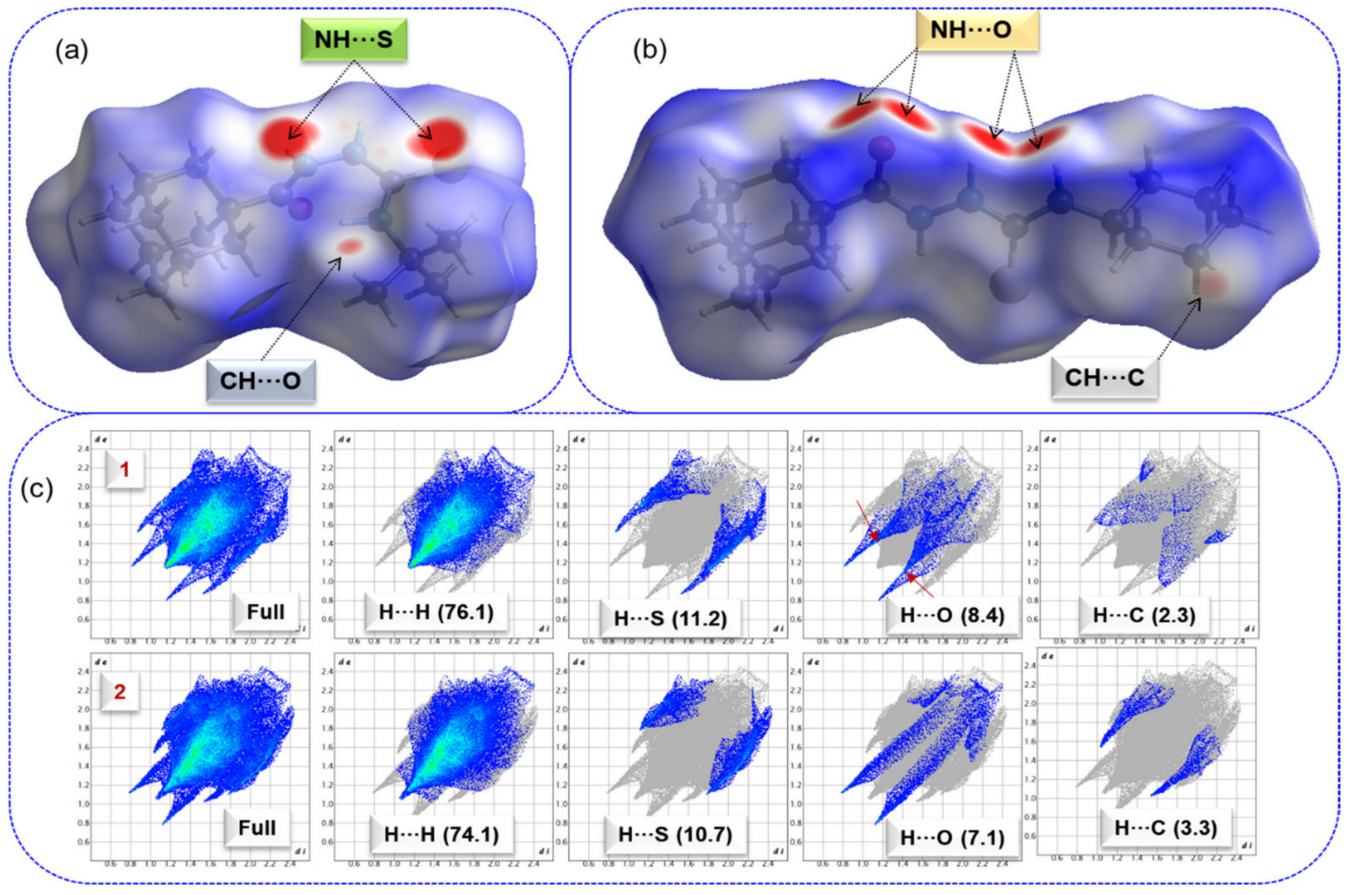
Hirshfeld surface shows the short intercontacts (red areas) observed in (**a**) compound **1**, (**b**) compound **2**, and (**c**) 2D-fingerprint plots for selected intercontacts, and their contributions are shown in parentheses. Arrow marks represent the intermolecular C–H···O interactions in **1**.

The intermolecular interactions observed in the crystal structures are qualitatively analyzed by using the Hirshfeld surface (HS) and 2D fingerprint plots. The HS and 2D-fingerprint plots were generated for compounds **1** and **2** to understand the effect of *tert*-butyl and cyclohexyl moieties on the intermolecular interactions. Figure 2 shows the HS over the normalized distance *d*_norm_ values for structures **1** and **2**.

In compound **1**, the broad and intense red spots correspond to short intermolecular N–H···S interactions that result in a centrosymmetric dimer, and a relatively small red spot near one of the methyl groups of the *tert*-butyl moiety represents a short C–H···O interaction (Figure 2a). In compound **2**, the large intense red spots result from a pair of N–H···O interactions, and a less intense red spot located near the cyclohexyl group belongs to a short C–H···C(π) interaction (Figure 2b). HS analysis indicates that these short intermolecular contacts play a significant role in stabilizing crystal packing.

Figure 2c shows the complete and decomposed 2D-FP plots for different intercontacts observed in these two structures. This figure also reveals differences in the inter-contact distribution patterns due to *tert*-butyl and cyclohexyl substituents. In both structures, the intermolecular H···H interactions occupy an overwhelming portion (>70%) of the HS area toward crystal packing and their relative contributions are comparable. The tip distance (*d*_i_ + *d*_e_) of H···H contact is located at approximately 2.2 Å in **1** and slightly lengthy (~2.3 Å) in **2**. The relative contribution of H···S interactions is also comparable in **1** and **2**. However, tip distance of H···S contacts reveals differences. In compound **1**, the corresponding distance is at 2.4 Å, confirming the presence of N–H···S interactions, while the corresponding distance is around 2.9 Å, confirming the absence of N–H···S interactions and the presence of C–H···S interactions in compound **2**. The relative contribution of H···O interactions is also comparable in compounds **1** and **2**. However, the differences in the distribution pattern of H···O contacts occurred. The tip distance is at approximately 1.9–2.0 Å, confirming the presence of N–H···O interactions in **2**. In **1**, the decomposed 2D-FP plot for H···O contacts reveals a pair of sharp spikes at ~2.5 Å, confirming the existence of C–H···O interactions. This feature is not visible for structure **2** and hence no C–H···O interactions in the solid state. Although the contribution of H···C contacts is relatively small, the tip distance for sharp spikes is located at 2.6 Å in **2**, confirming the possible existence of a C–H···C(π) interaction. The distribution pattern of this contact is not a typical wing-like pattern in **1**, resulting in the absence of such a contact in this structure.

#### 2.2.3. Molecular Dimers and Supramolecular Self-Assembly of Compound **1**

**Figure 3:**
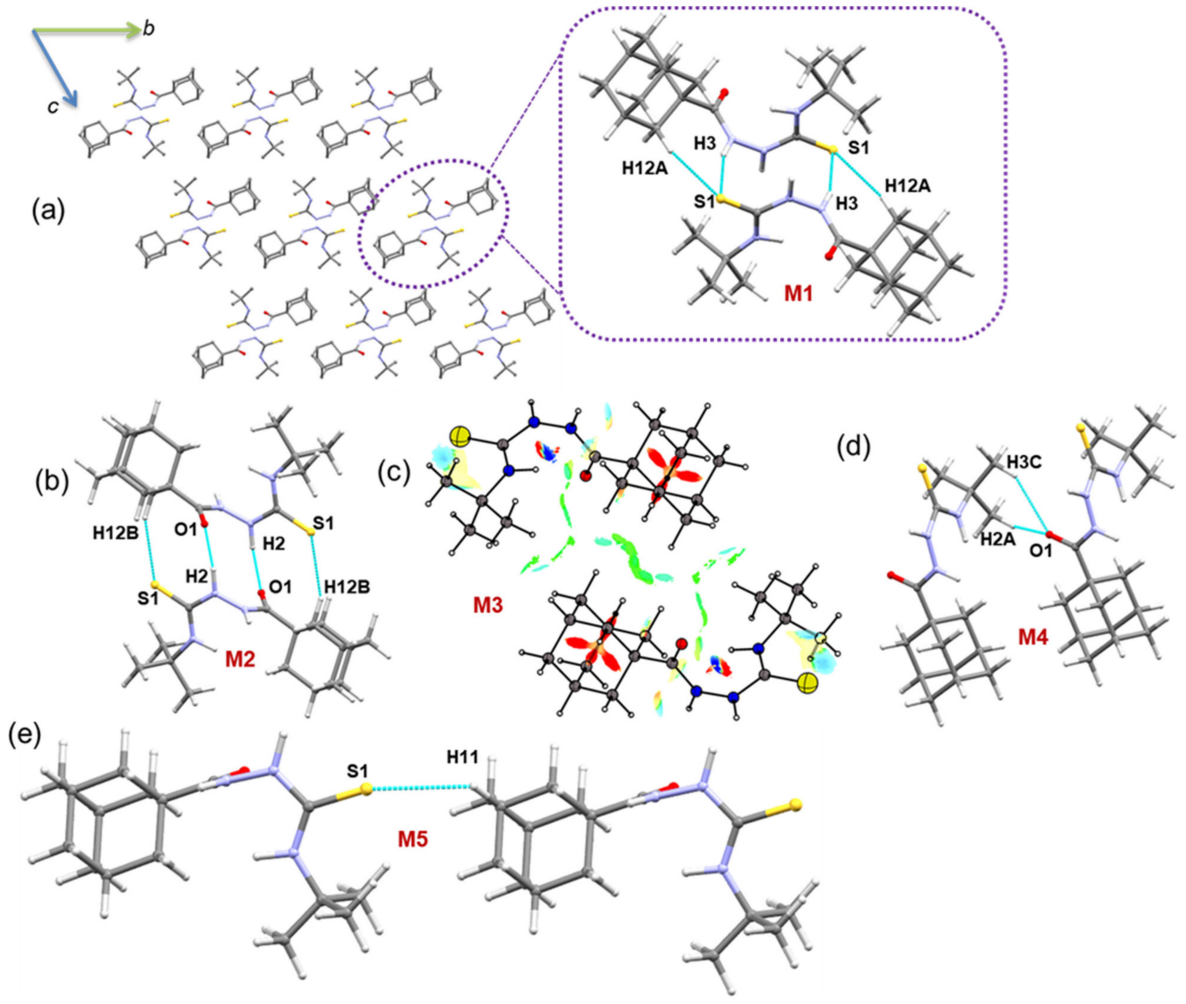
(**a**) Crystal packing of **1** and the primary structural motif M1 observed in this structure is shown (dashed box) and (**b**–**e**) other molecular dimers of **1**. The NCI plot is shown for molecular dimer M3.

In the solid state, molecules of compound **1** are packed in a columnar fashion along the crystallographic *bc* plane (Figure 3), and the solid-state structure is stabilized mainly by intermolecular N–H···S/O and C–H···S/O interactions. We carried out the CLP-PIXEL calculation to identify energetically significant dimers from the crystal structure of compound **1**. The result shows five molecular dimers with intermolecular interaction energies for these dimers (M1 to M5) ranging from −24.4 to −4.0 kcal mol^−1^. A quantum chemical calculation with the B97D3/def2-TZVP level of theory was used to obtain accurate interaction energies for these dimers as previously reported [53,54,55]. The complexation energies calculated by the PIXEL and DFT methods are comparable for the most of the dimers (Table 3).

As shown in Figure 3a, the primary motif (M1) generates a dimer that is stabilized by N/C–H···S interactions. The electrostatic energy component contributes approximately 65% toward the stabilization of dimer M1. The N–H···S interaction generates $R_{2}^{2}$(10) graph-set motif, whereas the C–H···S interaction produces the $R_{2}^{2}$(16) motif. When these two interactions are combined, the dimer M1 consists of $R_{1}^{2}$(7)-$R_{2}^{2}$(10)-$R_{1}^{2}$(7) ring motifs.

The second-most stabilized dimer is formed by N–H···O and C–H···S interactions and is slightly more electrostatic in nature (68%) than the dimer M1. When these interactions are considered individually, the former interaction generates an $R_{2}^{2}$(10) ring, whereas the latter interaction produces an $R_{2}^{2}$(16) ring (Figure 3b).

In dimer M3, there is no conventional interaction that could stabilize it. However, short H···H contacts established less than 2.4 Å between the H atoms of the *tert*-butyl group and the adamantyl moiety and between the adamantyl–adamantyl groups. The NCI plot shows the weak nature of stabilizing interactions by H···H short contacts (Figure 3c). The PIXEL energy analysis supports this weak nature of H···H contacts with 76% of the dispersion energy contributing to stabilizing this dimer. Two methyl groups of the tert-butyl moiety act as donors for the weak C–H···O=C interaction which forms dimer M4. For stabilization, the electrostatic and dispersion energy components contribute 30% and 70%, respectively. The least stable dimer M5 is formed by a weak C–H···S=C interaction that generates a molecular chain that runs parallel to the crystallographic *b* axis.

#### 2.2.4. Molecular Dimers and Supramolecular Features in Compound **2**

**Figure 4:**
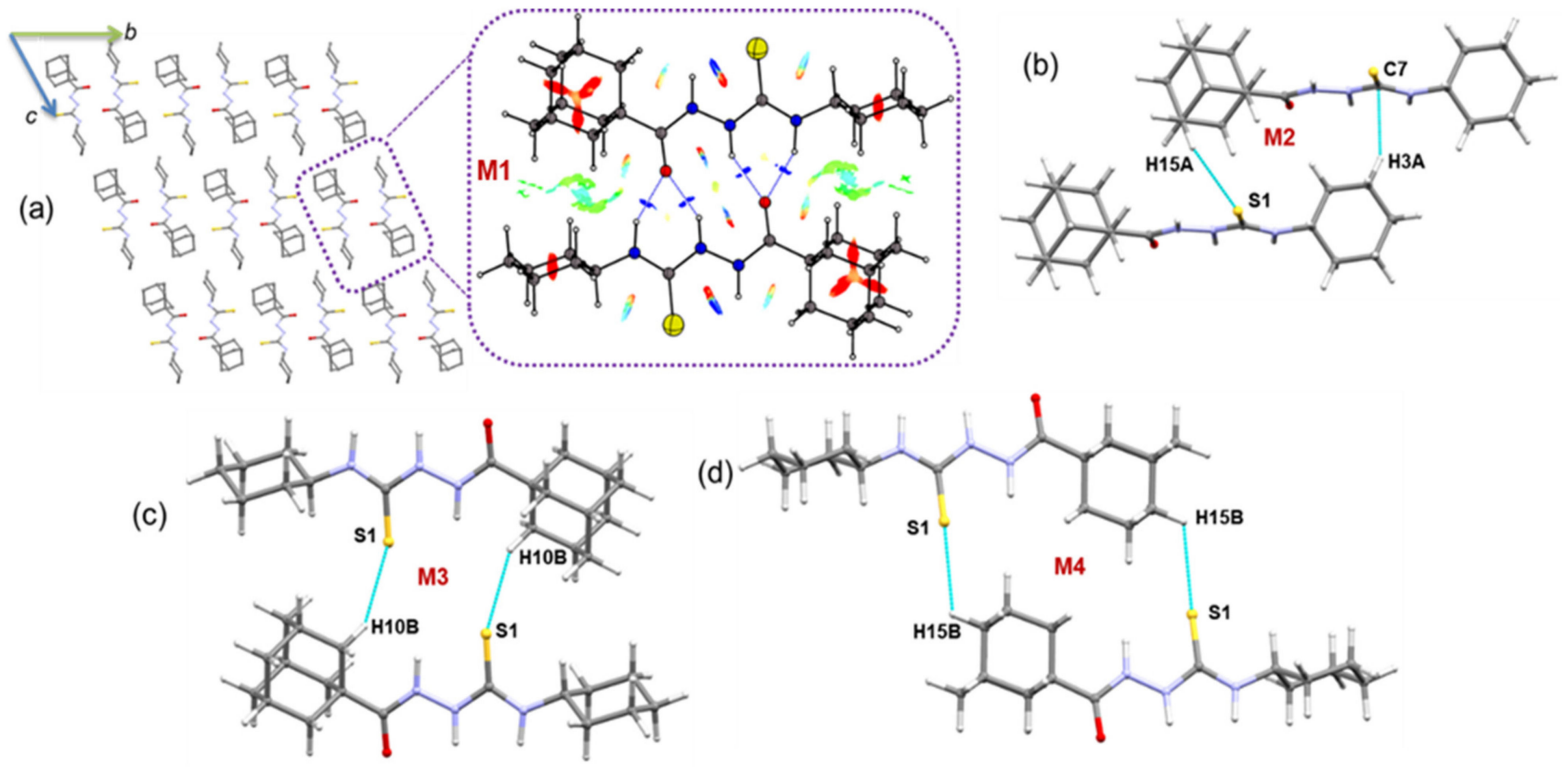
(**a**) Columnar packing of 2 and the NCI plot for the primary motif M1 formed in **2** is shown (dashed box) and (**b**–**d**) molecular dimers observed in the crystal structure of **2**.

Regardless of the *tert*-butyl/cyclohexyl substitutions, the crystal packing of compound **2** is somewhat similar to that of molecule **1**. In the crystalline state, molecules of compound **2** are also packed in a columnar fashion along the crystallographic *bc* plane (Figure 4), and the crystal structure is primarily stabilized by intermolecular N–H···O and C–H···S/C interactions.

The CLP-PIXEL calculation identifies four energetically significant dimers (M1 to M4) in compound **2**. The intermolecular interaction energies for these dimers range from −32.2 to −4.9 kcal mol^−1^. The complexation energies for the dimers calculated by the PIXEL and DFT methods are comparable. As shown in Figure 4a, the primary motif (M1) is formed by centrosymmetrically related molecules and is stabilized by a pair of N–H···O interactions. This dimer is predominantly electrostatic in nature, with a 74% contribution toward stabilization. The M1 motif has also been observed in closely related structures, namely *N*-(2-chlorophenyl)-2-(pyridin-2-ylcarbonyl)hydrazinecarbothioamide [56], 2-(2-methoxybenzoyl)-*N*-phenylhydrazinecarbothioamide, and 2-(2,5-dimethoxybenzoyl)-*N*-phenylhydrazinecarbothioamide [57], 2-(((3,5-di-t-butyl-4-hydroxybenzyl)sulfanyl)acetyl)-*N*-(3-fluorophenyl)hydrazinecarbothioamide [58], *N*-phenyl-2-[tricyclo[8.2.2.24,7]-hexadeca-1(12),4,6,10,13,15-hexaene-5-carbonyl]hydrazine-1-carbothioamide [59], -(4-chlorophenyl)-1-(2-hydroxy-2,2-diphenylacetyl)thiosemicarbazide [60], 2-(4-(5,5-difluoro-1,3,7,9-tetramethyl-5*H*-6l5,5l5-dipyrrolo[1,2-c:2′,1′-*f*][1-3]diazaborinin-10-yl)-benzoyl)-*N*-(4-nitrophenyl)hydrazinecarbothioamide [59]. In addition to N–H···O interactions, green patches (Figure 4a) were located between the cyclohexyl and adamantyl groups due to the short H···H contacts of these groups.

Dimer M2 is formed by intermolecular C–H···S interaction in which adamantyl H atom involved as a donor. In addition to this interaction, one of the cyclohexane H atoms is involved as a donor for a short C–H···C(π)=S interaction with the H···C separation of 2.58 Å. For the stabilization, the dispersion energy component contributes approximately 70%. The remaining two dimers (M3 and M4) have stabilized by intermolecular C–H···S interactions and each of this dimer forming a cyclic dimer with $R_{2}^{2}$(16) [motif M3) and $R_{2}^{2}$(20) [motif M4]. Furthermore, the H···S distance is slightly shorter in dimer M3 than dimer M4. The close H···S contact is also reflected in the stabilization energy. In the former dimer, the electrostatic energy contributes 36% whereas the corresponding energy contribution is slightly reduced (30%) toward stabilization of the latter dimer.

#### 2.2.5. Lattice Energy and Energy Frameworks

The lattice energies for crystal structures **1** and **2** were calculated by using the AA-CLP-PIXEL method. The total lattice energy and its components, such as Coulombic, polarization, dispersion, and repulsion terms, are presented in Table 4. The results of lattice energy calculation suggest that crystal structure **2** has higher stabilization energy by 3 kcal mol^−1^ compared to crystal structure **1**. The repulsion term is comparable in both crystals, and slight variations are noted in the electrostatic and dispersion energy components. We also note that the contribution of electrostatic energy is nearly the same for **1** (48%) and **2** (47%) toward stabilization. The contribution of dispersion energy is slightly higher compared to electrostatic energy. However, its contribution is nearly the same in structures **1** (52%) and **2** (53%).

**Figure 5:**
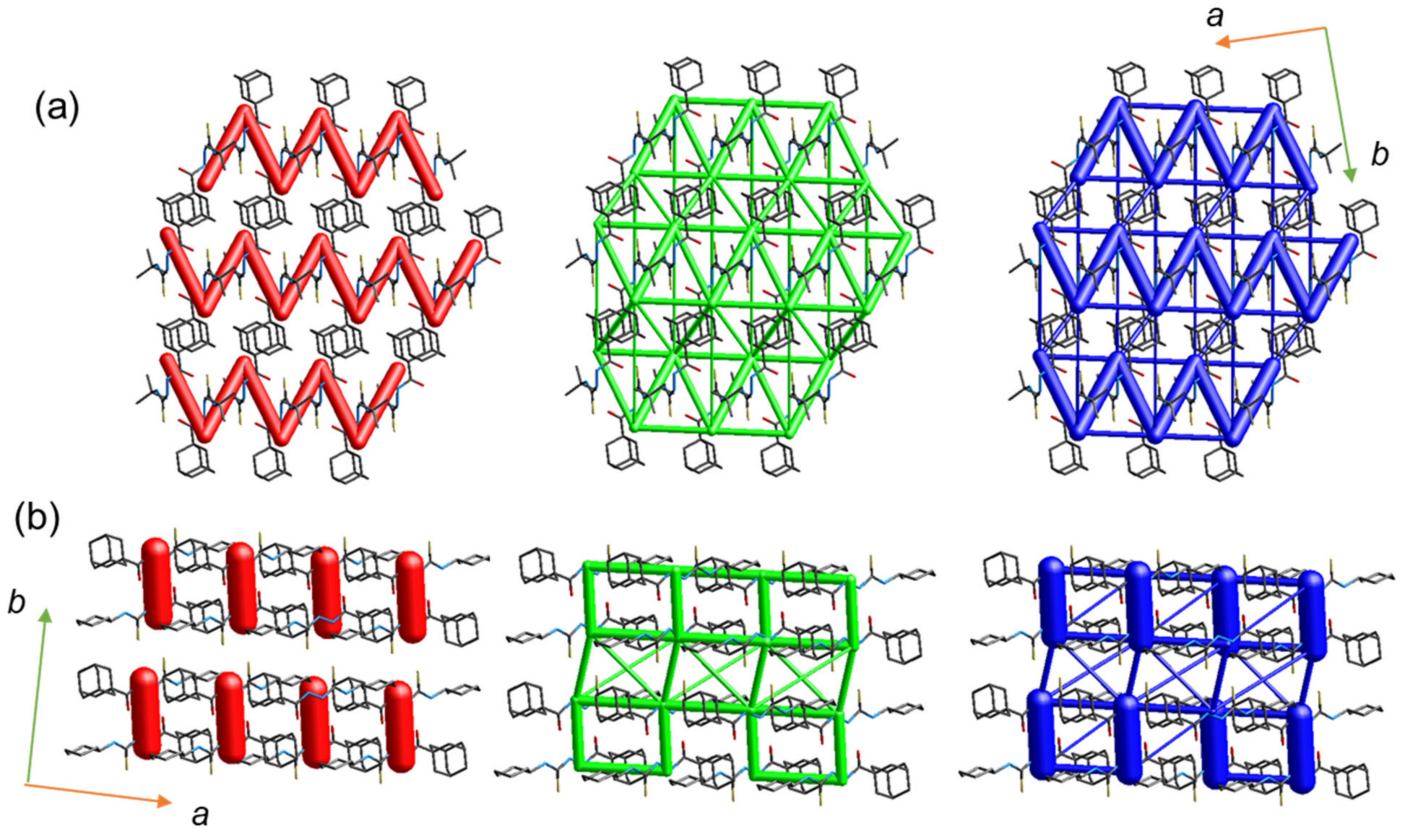
Energy frameworks for the crystal structure of (**a**) **1** and (**b**) **2**. The electrostatic (red), dispersion (green) and total interaction (blue) energies are represented as cylindrical tubes with size 80. The interaction energies with magnitudes smaller than −15 kJ mol^−1^ have been omitted for clarity.

As mentioned earlier, both molecules are packed in a columnar fashion in the crystalline state. We performed energy frameworks for the crystal structures to visualize the 3D topology of the energy frameworks. As shown in Figure 5, the energy frameworks offer similar and dissimilar features. Layers of vertical cylinders (electrostatic) are generated that are parallel to the crystallographic *b* axis for compound **2**, whereas zigzag layers run along the crystallographic *a* axis for compound **1**. The formation layers in both structures are due to the columnar packing. The zigzag layers in compound **1** correspond to alternate motifs formed by intermolecular N–H···S (D1) and C–H···O (d4) interactions. The zigzag layers are not presented due to the absence of C–H···O interaction in compound **2**. In compound **1**, the 3D topology of the dispersion energy component displays a complex ladder-like pattern, whereas in compound **2**, it shows a simple ladder-like structure. A small cylindrical tube in the dispersion energy framework corresponds to the short H···H contacts between the *tert*-butyl-adamantane groups in **1** and between the adamantane–adamantane and cyclohexane–adamantane groups in compound **2**. Differences in the 3D energy frameworks for crystals **1** and **2** suggest different mechanical behaviors at the molecular level.

#### 2.2.6. QTAIM Topological Features of Intermolecular Interactions

The topological analysis of intermolecular interactions observed in different dimers of compounds **1** and **2** was carried out within the framework of QTAIM to quantify the strength and nature of these interactions. The molecular graphs for all dimers observed in structures **1** and **2** are shown in Figures S1 and S2 (Supplementary Materials). The topological parameters, including electron density *ρ*(r), the Laplacian of electron density ∇^2^*ρ*(r), potential electronic energy density *V*(r), kinetic electronic energy density *G*(r), and bond path *R*_ij_, at the bond critical points for intermolecular interactions are summarized in Table 5.

The topological analysis shows positive values for the total electronic density (*H*(r)), the Laplacian of the electron density, and the ratio of $\left|\frac{-V(r)}{G(r)}\right|$ < 1 for all intermolecular interactions observed in structures **1** and **2**. This condition suggests that all interactions are closed-shell in nature, including the N–H···O interactions. In **1**, the N–H···O is found to be the strongest interaction among other interactions, and this interaction generates the primary motif in the solid state. The dissociation energy value (*D*_e_) for this interaction is 5.2 kcal mol^−1^. The next-strongest interaction is the N–H···S interaction with the *D*_e_ value of 3.3 kcal mol^−1^. The strength of all three C–H···S interactions is nearly the same. In addition, the strength of one of the C–H···O interactions (*D*_e_ = 1.5 kcal mol^−1^) is slightly higher in comparison with C–H···S interactions, and the remaining one is found to be almost the same.

Moreover, in structure **2**, the strength of intermolecular N–H···O interaction (*D*_e_ = 6.3–6.9 kcal mol^−1^) is strong compared to other interactions observed. However, these interactions are slightly stronger than those observed in structure **1**. It is of interest to note that C–H···S interactions strengths are very similar in both structures. We also note that intermolecular C–H···C(π) interaction is slightly stronger (*D*_e_ = 1.6 kcal mol^−1^) compared to C–H···S interactions.

### 2.3. In Vitro Urease Inhibitory Activity

The in vitro urease inhibitory activity of compounds **1** and **2** against Canavalia ensiformis (Jack bean) urease was evaluated by using the previously described indophenol method [61,62]. The results of the in vitro urease inhibition activity of compounds **1**, **2** and the standard urease inhibitor thiourea are shown in Table 6. According to the results of urease inhibitory activity, compounds **1** and **2** exhibited potent activity with IC_50_ values of 1.20 and 2.44 μM, respectively, representing 17.72 and 8.71 times the activity of thiourea. Consequently, compounds **1** and **2** would be considered as promising urease inhibitory hits for further investigations.

### 2.4. In Vitro Antiproliferative Activity

The in vitro antiproliferative activity of compounds **1** and **2** was assessed against five human tumor cell lines, namely human prostate cancer (PC-3), human colorectal cancer (HCT-116), human hepatocellular cancer (HepG-2), human cervical cancer (HeLa) and human breast cancer (MCF) by using the 3-[4,5-dimethylthiazoyl-2-yl]-2,5-diphenyltetrazolium bromide (MTT) colorimetric assay [63]. The results of in vitro antiproliferative activity of compounds **1** and **2** and the potent anticancer drug doxorubicin [64] are shown in Table 7. According to the antiproliferative activity results, compounds **1** and **2** displayed potent antiproliferative activity (IC_50_ < 10 μM) against HeLa and MCF-7 cell lines. The compounds were moderately active against HCT-116 and HepG-2 cell lines and weakly active against PC-3 cell lines. Based on the antiproliferative activity of compounds **1** and **2**, it would be considered as a promising anticancer drug candidate for further investigations.

### 2.5. Molecular Docking Analysis

**Figure 6:**
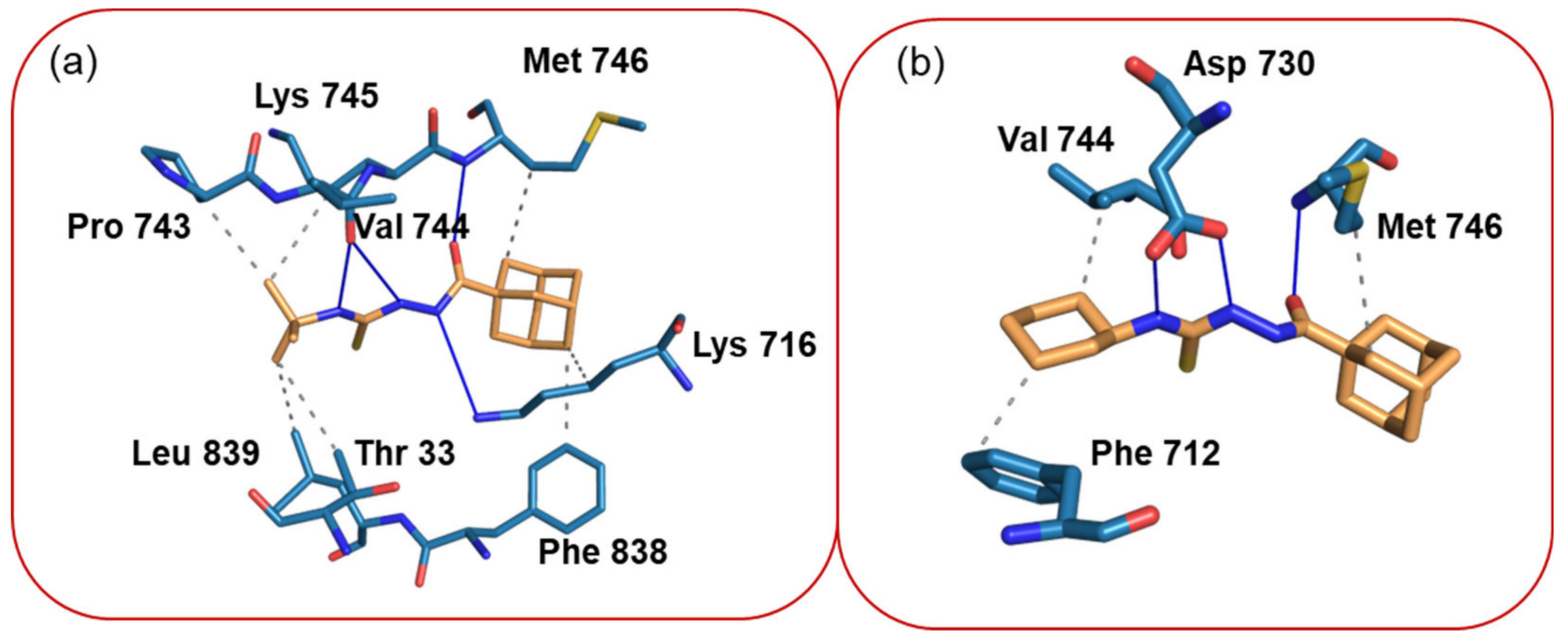
(**a**) Interactions between compound **1** and active residues of the urease enzyme and (**b**) intermolecular interactions between compound **2** and active site residues of the urease enzyme.

To examine binding pose of the title compounds and their interactions with the active site of the urease enzyme, we carried out molecular docking analysis by using the XP glide scoring scheme as mentioned in the experimental section. The molecular docking analysis reveals that both compounds bind better than thiourea at the active site of the urease (Table 8). Additionally, the docked poses were refined by using the MM-GBSA approach and the binding free energy (ΔG_bind_) of the ligands was calculated. The free energy calculation suggests that the binding affinity of compound **1** is slightly higher than compound **2**, which is in good agreement with the in vitro experimental data. The refined complexes were used to analyze the protein–ligand interaction by using a PLIP web server, as mentioned in the experimental section. As shown in Figure 6, all amine groups in compound **1** establish hydrogen bonding interactions with the O atom of the backbone of Val 744 and the N atom of the side chain Lys 716. The carbonyl O atom of the amide group of the compound **1** is involved in a hydrogen bond interaction with the N atom of the backbone of the Met 746 residue (Figure 6a). The side chains of the residues Lys 716, Met 746, and Phe 838 participate in hydrophobic interactions with the adamantane moiety of compound **1**. Similarly, the *tert*-butyl group also participates in hydrophobic interactions with the side chains of the residues Thr 33, Lys 745, Pro 743, and Leu 839.

The slightly lower binding affinity of compound **2** to the urease enzyme could be understood from the fewer interactions formed between this compound and the enzyme. Only two amine groups of two are involved in hydrogen bonding with the active residue Asp 730. The carbonyl O atom of the ligand establishes a hydrogen bond with the N atom of the backbone of Met 746 as observed in **1**. Met 746 also participates in a hydrophobic interaction with the adamantane moiety of **2**. Similarly, the cyclohexyl moiety establishes hydrophobic contacts with the residues Phe 712 and Val 744 (Figure 6b).

## 3. Materials and Methods

### 3.1. Synthesis and Crystallization

To a solution of adamantane-1-carbohydrazide **C** (1.95 g, 0.01 mol) in ethanol (10 mL), *t*-butyl isothiocyanate or cyclohexyl isothiocyanate (0.01 mol) was added and the reaction mixture was heated under reflux with stirring for 30 min. Upon cooling, the precipitated crude products were filtered, washed with cold ethanol, dried and recrystallized from ethanol to the target compounds **1** and **2** (Scheme 1). ^1^H- and ^13^C NMR spectra are shown in Supplementary Materials (Figures S3–S6).

#### 3.1.1. 2-(Adamantane-1-carbonyl)-*N*-(*tert*-butyl)hydrazine-1-carbothioamide **1**

Colorless rectangular prism crystals. Yield 2.85 g (92%); Mp. 190–192 °C. ^1^H NMR (DMSO-*d*_6_, 500.16 MHz): *δ* 1.40 (s, 9H, CH_3_), 1.61–1.68 (m, 6H, Adamantane-H), 1.80 (s, 6H, Adamantane-H), 1.95 (s, 3H, Adamantane-H), 6.22 (s, 1H, NH), 8.93 (s, 1H, NH), 9.42 (s, 1H, NH). ^13^C NMR (DMSO-*d*_6_, 125.77 MHz): *δ* 28.64, 52.64 (*t*-Butyl-C), 27.53, 36.03, 38.36, 39.50 (Adamantane-C), 176.10 (C=O), 180.09 (C=S). Analysis for C_16_H_27_N_3_OS (309.47): C, 62.04 (Calc. 62.10); H, 8.82 (Calc. 8.79); N, 13.83 (Calc. 13.58); S, 10.35 (Calc. 10.36).

#### 3.1.2. 2-(Adamantane-1-carbonyl)-*N*-cyclohexylhydrazine-1-carbothioamide **2**

Colorless rectangular prism crystals. Yield 3.22 g (96%); Mp. 235–237 °C. ^1^H NMR (DMSO-*d*_6_, 500.16 MHz): *δ* 1.11–1.15 (m, 1H, Cyclohexane-H), 1.23–1.29 (m, 4H, Cyclohexane-H), 1.52–1.55 (m, 1H, Cyclohexane-H), 1.62–1.68 (m, 8H, Adamantane-H & Cyclohexane-H), 1.81–1.82 (m, 8H, Adamantane-H & Cyclohexane-H), 1.96 (s, 3H, Adamantane-H), 6.77 (s, 1H, NH), 8.98 (s, 1H, NH), 9.34 (s, 1H, NH). ^13^C NMR (DMSO-*d*_6_, 125.77 MHz): *δ* 25.53, 25.14, 31.85, 52.28 (Cyclohexane-C), 27.51, 36.07, 38.18, 39.50 (Adamantane-C), 176.25 (C=O), 181.0 (C=S). Analysis for C_18_H_29_N_3_OS (335.51): C, 64.32 (Calc. 64.44); H, 8.80 (Calc. 8.71); N, 12.38 (Calc. 12.52); S, 9.58 (Calc. 9.56).

### 3.2. Single Crystal X-ray Diffraction (SCXRD)

Single-crystal X-ray diffraction data were collected at 160(1) K on a Rigaku OD XtaLAB Synergy, Dualflex Pilatus 200K diffractormeter (compound **1**) and Rigaku OD SuperNova/Atlas area-detector diffractometer (compound **2**). For both cases, a single wavelength X-ray source (Cu Kα radiation: λ = 1.54184 Å) from a microfocus sealed X-ray tube and an Oxford liquid-nitrogen Cryostream cooler were used. The selected suitable single crystal was mounted by using polybutene oil on a flexible loop fixed on a goniometer head and immediately transferred to the diffractometer. Prior to the experiment, data collection, data reduction, and analytical absorption correction [65] were performed with the program suite *CrysAlisPro* (*CrysAlisPro* (version 1.171.40.68a), Rigaku Oxford Diffraction Ltd., Yarnton, Oxfordshire, England, 2019). By using *Olex2* [66], the structure was solved with the SHELXT [67] small molecule structure solution program and refined with the *SHELXL2018/3* program package [68] by full-matrix least-squares minimization on F^2^. *PLATON* [69] was used to check the result of the X-ray analysis.

### 3.3. Hirshfeld Surface and Energy Frameworks

The Hirshfeld surface, the decomposed 2D fingerprint plots and the energy frameworks were calculated by using the CrystalExplorer-17.5 program [70]. The energy frameworks for title compounds were constructed by using the energies for various dimeric pairs observed in the crystal structure with B3LYP/6-31G(d,p) level of approximation [71,72].

### 3.4. PIXEL Energy Analysis

The intermolecular interaction energies for various molecular dimers were observed in the crystal structures of the title compounds by using the CLP-PIXEL program [73,74]. Electron density was obtained with the MP2/6-31G** levels of theory by using the Gaussian 09 program [75], and this calculated density was used for the calculation of the CLP-PIXEL energy.

### 3.5. DFT Calculations

Furthermore, the accurate complexation energies of the molecular pairs identified from the CLP-PIXEL energy analysis were calculated by using the B97D3/def2-TZVP level of theory, and then, these complexation energies were corrected (Δ*E*_cp_) for basis set superposition error by using the counterpoise method [76].

### 3.6. Topological Analysis

The topological analysis of selected dimers was performed within the framework of Bader’s quantum theory of atoms in molecules approach (QTAIM) by using the AIMALL program [77]. The wave functions were computed at the M062X-D3/def2-TZVP level of theory for topological analysis. The noncovalent interaction plot (NCIplot) index was also used to characterize the nature of noncovalent interactions [78].

### 3.7. Molecular Docking Analysis

The molecular docking study was performed via the Schrodinger suite (www.schrodinger.com, accessed on 20 October 2022) to predict the binding mode of the title compounds and thiourea in the active site of the Jack bean urease enzyme. The urease structure (PDB ID: 3LA4) was retrieved from the protein databank. The grid box was constructed based on the active site residues (Leu 13, Ala 16, Tyr 32, Val 36, Ala 37, Phe 712, Pro 717, Pro 743, Val 744, Met 746 and Leu 839) suggested earlier [79]. The glide XP scoring scheme [80] was used to predict the binding pose followed by the MM-GBSA approach to calculate the relative binding free energies of the ligands [81]. In the MM-GBSA free energy calculation, residues within 6 Å from the ligand and ligand molecule itself were treated as flexible during the minimization as suggested in our earlier work [82]. Protein–ligand interaction was analyzed by using the PLIP web tool [83].

### 3.8. Determination of In Vitro Urease Inhibitory and Antiproliferative Activities

The previously described indophenol method [61,62] and MTT colorimetric assay [63] were used for determination of in vitro urease inhibitory and antiproliferative activities, respectively (Supplementary Materials).

## 4. Conclusions

Potential adamantane-based urease inhibitors, namely (2-(Adamantane-1-carbonyl)-*N*-(*tert*-butyl)hydrazine-1-carbothioamide), **1**, and (2-(Adamantane-1-carbonyl)-*N*-cyclohexylhydrazine-1-carbothioamide), **2** have been synthesized, and crystal structures were analyzed in detail. These two compounds exhibit different conformations (folded and extended) in the solid state. The DFT calculations suggest that energy barriers exist between the folded and extended conformations. The Hirshfeld surface revealed that the *tert*-butyl and cyclohexyl moieties do not affect the contribution of the major intermolecular interactions in the solid state. However, they prefer to form different supramolecular motifs in the solid state. The energetics of molecular dimers were calculated by using the CLP–Pixel method. These molecular dimers stabilized by N–H···S, N–H···O, C–H···S, C–H···O, H-H bonding, and C–H···π interactions. Compound **2** does not prefer to form a N–H···S intermolecular interaction. Similarly, compound **1** does not like to form a C–H···π intermolecular interaction. The lattice energy calculation suggests that crystal structure **2** has higher stabilization energy than crystal structure **1**. The QTAIM analysis inferred that N–H···O interaction was found to be stronger compared to other interactions observed in these structures. We described the in vitro urease inhibition and antiproliferative activities of **1** and **2**. The results suggest that both compounds showed better binding ability in comparison to thiourea and showed moderate activity against different cancer cell lines. The molecular docking analysis revealed the binding modes of compounds **1** and **2** and their interactions with the key active site residues.

## Figures and Tables

**Table 1:** Crystallographic data and structure refinement parameters of compounds **1** and **2**.

	Compound 1	Compound 2
Empirical formula	C_16_H_27_N_3_OS	C_18_H_29_N_3_OS
Formula weight	309.46	335.50
Temperature (K)	160 (1)	160 (1)
Crystal system	Triclinic	Triclinic
Space group	P-1	P-1
a/Å	6.3890 (3)	6.9315 (3)
b/Å	11.8264 (7)	11.4885 (3)
c/Å	12.3792 (7)	12.7719 (5)
α/°	62.466(6)	65.012(4)
β/°	80.222 (5)	75.565 (3)
γ/°	87.511 (5)	82.280 (3)
Volume/Å^3^	816.71 (9)	892.27 (6)
Z	2	2
ρcalcg/cm^3^	1.258	1.249
μ/mm^−1^	1.775	1.665
F(000)	336.0	364.0
Crystal size/mm^3^	0.17 × 0.08 × 0.06	0.3 × 0.23 × 0.12
Radiation	Cu Kα (λ = 1.54184)	CuKα (λ = 1.54184)
2Θ range for data collection/°	8.172 to 154.456	7.818 to 148.95
Index ranges	−8 ≤ h ≤ 8, −14 ≤ k ≤ 11, −15 ≤ l ≤ 15	−8 ≤ h ≤ 8, −14 ≤ k ≤ 14, −15 ≤ l ≤ 12
Reflections collected	17,204	15,479
Independent reflections	3439 [R_int_ = 0.0251, R_sigma_ = 0.0189]	3633 [R_int_ = 0.0191, R_sigma_ = 0.0156]
Data/restraints/parameters	3439/0/205	3633/0/220
Goodness-of-fit on F^2^	1.019	1.012
Final R indexes [I> = 2σ (I)]	R_1_ = 0.0291, wR_2_ = 0.0784	R_1_ = 0.0348, wR_2_ = 0.0907
Final R indexes [all data]	R_1_ = 0.0307, wR_2_ = 0.0799	R_1_ = 0.0360, wR_2_ = 0.0915
Largest diff. peak/hole/e Å^−3^	0.27/−0.20	0.27/−0.27
CCDC No.	2,053,083	2,215,239

**Table 2:** Selected bond lengths in **1** and **2**. The bonds in bold corresponds to structure **2**.

Bond	Compound 1	Conformer 1a	Compound 2	Conformer 2a
C7–C6/**C9–C8**	1.527	1.517	1.518	1.524
C6=O1/**C8=O1**	1.219	1.223	1.241	1.206
C6–N3/**C8–N3**	1.365	1.349	1.323	1.384
N3–N2/**N3–N2**	1.389	1.376	1.385	1.379
N2–C5/**N2–C7**	1.361	1.356	1.354	1.394
C5=S1/**C7=S1**	1.690	1.676	1.677	1.661
C5–N1/**C7–N1**	1.331	1.348	1.336	1.334
N1–C1/**N1–C1**	1.486	1.477	1.458	1.465

**Table 3:** Intermolecular interaction energies (in kcal mol^−1^) for different dimers were obtained from the crystal structures of **1** and **2** by using the CLP-PIXEL method. The BSSE-corrected dimerization energies calculated by the DFT method were given for comparison.

Dimer	CD	Symmetry	Important Interactions	Geometry^a^ H···A (Å), ∠D–H···A (°)	PIXEL/MP2/6-31G**					B97D3/def2-tzvp
					*E*_Coul_	*E*_pol_	*E*_disp_	*E*_rep_	*E*_tot_	Δ*E*_cp_
**Compound 1**										
M1	6.289	−x + 1, −y + 1, −z + 1	N3–H3···S1	2.39, 163	−20.8	−13.6	−18.6	28.7	−24.4	−26.7
			C12–H12A···S1	2.87, 169						
M2	6.583	−x + 2, −y + 1, −z + 1	N2–H2···O1	1.97, 173	−19.3	−8.5	−12.9	21.7	−19.0	−19.2
			C12–H12B···S1	2.92, 146						
M3	7.182	−x + 2, −y + 2, −z	Adamantane···Adamantane and *t*-butyl···adamanatane [H-H bonding]	<2.40	−1.6	−1.1	−8.4	5.0	−6.2	−7.5
M4	6.389	x−1, y, z	C2–H2A···O1	2.45, 156	−1.9	−1.8	−8.7	6.5	−5.9	−7.3
			C3–H3C···O1	2.69, 146						
M5	11.826	x, y−1, z	C11–H11···S1	2.95, 126	−2.2	−1.2	−5.0	4.4	−4.0	−4.6
**Compound 2**										
M1	5.563	−x + 1, y + 1, −z + 1	N1–H1A···O1	1.95, 152	−36.6	−15.1	−18.2	37.7	−32.2	−30.6
			N2–H2···O1	1.91, 148						
M2	6.931	x−1, y, z	C15–H15A···S1	2.98, 166	−3.9	−2.2	−14.0	10.3	−9.8	−11.8
			C3–H3A···C7	2.58, 151						
M3	6.036	−x + 1, −y, −z + 1	C10–H10B···S1	2.89, 152	−3.2	−2.6	−10.3	7.9	−8.2	−9.6
M4	7.924	−x + 2, −y, −z + 1	C15–H15B···S1	3.01, 115	−2.0	−1.2	−7.5	5.8	−4.9	−6.4

**Table 4:** Lattice energy for crystal structures **1** and **2**. Energy is expressed in kcal mol^−1^.

Compound	*E*_Coul_	*E*_pol_	*E*_disp_	*E*_rep_	*E*_tot_
**1**	−25.6	−14.2	−43.7	43.3	−40.2
**2**	−27.2	−13.0	−46.2	43.2	−43.2

**Table 5:** Topological parameters for selected intermolecular interactions in different dimers of compounds **1** and **2** at their (3, −1) BCPs. Definitions: *R*_ij_, bond path (Å); *ρ*(r), electron density (e Å^−3^); ∇^2^*ρ*(r), Laplacian of electron density (e Å^−5^); *V*(r), potential electron density (kJ mol^−1^ br^−3^); *G*(r), kinetic electron density (kJ mol^−1^ br^−3^); *H*(r), total electronic energy density (kJ mol^−1^ br^−3^); *D*_e_, dissociation energy (kcal mol^−1^).

Interaction	*R*_ij_	*ρ*(r)	∇^2^*ρ*(r)	*V*(r)	*G*(r)	*H*(r)	$\left|\frac{-V(r)}{G(r)}\right|$	*D_e_*
**Compound 1**								
D1								
N3–H3···S1	2.416	0.129	1.253	−27.4	30.7	3.4	0.89	3.3
C12–H12A···S1	2.900	0.059	0.546	−10.7	12.8	2.1	0.84	1.3
D2								
N2–H2···O1	1.995	0.146	2.169	−43.5	51.3	7.8	0.85	5.2
C12–H12B···S1	2.961	0.049	0.484	−8.7	10.9	2.2	0.80	1.0
D4								
C2–H2A···O1	2.464	0.057	0.807	−12.7	17.4	4.6	0.73	1.5
C3–H3C···O1	2.715	0.036	0.480	−7.4	10.3	2.8	0.73	0.9
D5								
C11–H11···S1	3.014	0.042	0.484	−8.4	10.8	2.4	0.78	1.0
**Compound 2**								
D1								
N2–H2···O1	1.934	0.170	2.650	−52.6	64.9	7.3	0.89	6.9
N1–H1A···O1	1.970	0.162	2.474	−52.3	59.9	7.6	0.87	6.3
D2								
C15–H15A···S1	2.996	0.049	0.436	−8.4	10.1	1.8	0.83	1.0
C3–H3A···C7	2.603	0.056	0.665	−12.9	15.5	2.6	0.83	1.6
D3								
C10–H10B···S1	2.926	0.046	0.477	−8.4	10.7	2.3	0.79	1.0
D4								
C10–H10B···S1	2.926	0.046	0.477	−8.4	10.7	2.3	0.79	1.0

**Table 6:** In vitro urease inhibitory activity of compounds **1** and **2**.

Compound	IC_50_ ± SEM (μM) *
**1**	1.20 ± 0.07
**2**	2.44 ± 0.11
**Thiourea**	21.26 ± 0.12

* Values are the means of three determinations ± Standard error mean (SEM).

**Table 7:** In vitro anti-proliferative activity of compounds **1** and **2**.

Compound	IC_50_ (µM)				
	PC-3	HCT-116	HepG-2	HeLa	MCF-7
**1**	48.50 ± 2.9	28.69 ± 2.0	26.12 ± 1.6	**7.82 ± 0.6**	**13.20 ± 0.4**
**2**	52.68 ± 4.3	34.90 ± 2.8	29.48 ± 1.4	**9.88 ± 1.0**	**16.98 ± 0.6**
**Doxorubicin**	**8.87 ± 0.6**	**5.23 ± 0.3**	**4.17 ± 0.2**	**4.17 ± 0.2**	**5.57 ± 0.4**

**Table 8:** Molecular docking score and binding free energy for compounds **1** and **2**.

Compound	Glide XP Score (in kcal mol^−1^)	ΔGbind (MM-GBSA) (in kcal mol^−1^)
**1**	−3.287	−36.90
**2**	−3.939	−26.03
**Thiourea**	−2.486	−4.34

## Data Availability

The supplementary crystallographic data could be obtained free of charge from the Cambridge Crystallographic Data Centre (www.ccdc.cam.ac.uk/data_request/cif) using the accession numbers, CCDC-2053083 (compound **1**) and CCDC-2215239 (compound **2**).

## Supplementary Materials

The following supporting information can be downloaded at: https://www.mdpi.com/article/10.3390/molecules27238425/s1, Figure S1: Molecular graphs of various dimers observed in compound **1** showing important intermolecular interactions at their bond critical points; Figure S2: Molecular graphs of various dimers observed in compound **2** showing important intermolecular interactions at their bond critical; Figure S3: ^1^H NMR spectrum of compound **1** in DMSO-*d*_6_; Figure S4: ^13^C NMR spectrum of compound **1** in DMSO-*d*_6_; Figure S5: ^1^H NMR spectrum of compound **2** in DMSO-*d*_6_; and Figure S6: ^13^C NMR spectrum of compound **2** in DMSO-*d*_6_, the experimental details for determination of in vitro urease inhibitory and antiproliferative activities.

## Sample Availability

Samples of compounds **1** and **2** are available from the authors.
