# Supplementary material for: X-ray Structures and Computational Studies of Two Bioactive 2-(Adamantane-1-carbonyl)-N-substituted Hydrazine-1-carbothioamides
Source: Molecules. 2022 Dec 1;27(23):8425. doi: 10.3390/molecules27238425 (PMC9741201; doi:10.3390/molecules27238425)
Supplement: Supplementary file 1 [file molecules-27-08425-s001.zip › molecules-2028018-supplementary.pdf]

Supplementary Material

X-Ray Structures and Computational Studies of Two Bioactive 2-(Adamantane-1-carbonyl)-*N*-substituted Hydrazine-1-carbothioamides

Lamya H. Al-Wahaibi [1], Kowsalya Alagappan [2], Olivier Blacque [3], Ahmed A. B. Mohamed [4], Hanan M. Hassan [5], María Judith Percino [6], Ali A. El-Emam [4,*] and Subbiah Thamotharan [2,*]

1 Department of Chemistry, College of Sciences, Princess Nourah bint Abdulrahman University, Riyadh 11671, Saudi Arabia
2 Biomolecular Crystallography Laboratory, Department of Bioinformatics, School of Chemical and Biotechnology, SASTRA Deemed University, Thanjavur 613 401, India
3 Department of Chemistry, University of Zurich, Winterthurerstrasse 190, 8057 Zurich, Switzerland
4 Department of Medicinal Chemistry, Faculty of Pharmacy, Mansoura University, Mansoura 35516, Egypt
5 Department of Pharmacology and Biochemistry, Faculty of Pharmacy, Delta University for Science and Technology, International Costal Road, Gamasa City, Mansoura 11152, Egypt
6 Unidad de Polímeros y Electrónica Orgánica, Instituto de Ciencias, Benemérita Universidad Autónoma de Puebla, Val3-Ecocampus Valsequillo, Independencia O2 Sur 50, San Pedro Zacachimalpa, Puebla 72960, CP, Mexico
* Correspondence: elemam@mans.edu.eg (A.A.E.-E.); thamu@scbt.sastra.edu (S.T.); Tel.: +20-50-2258087 (A.A.E.-E.)

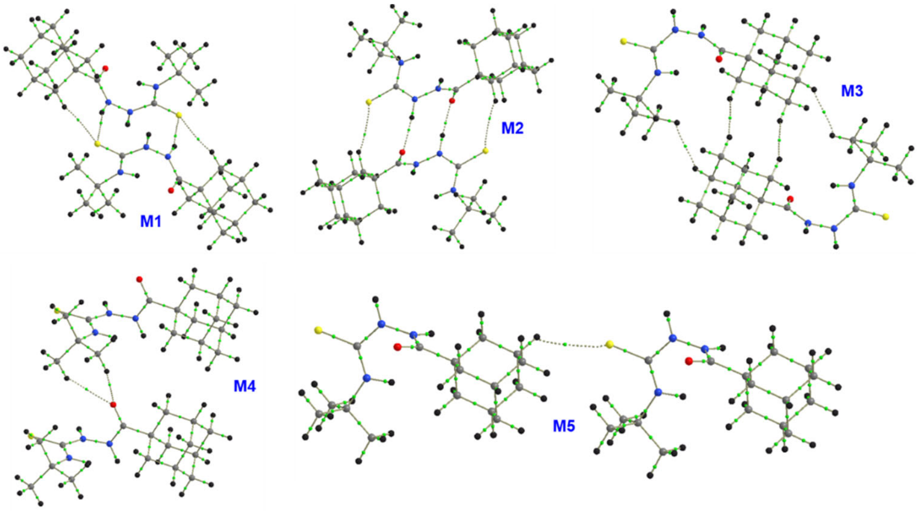

Figure S1. Molecular graphs of various dimers observed in compound **1** showing important intermolecular interactions at their bond critical points.

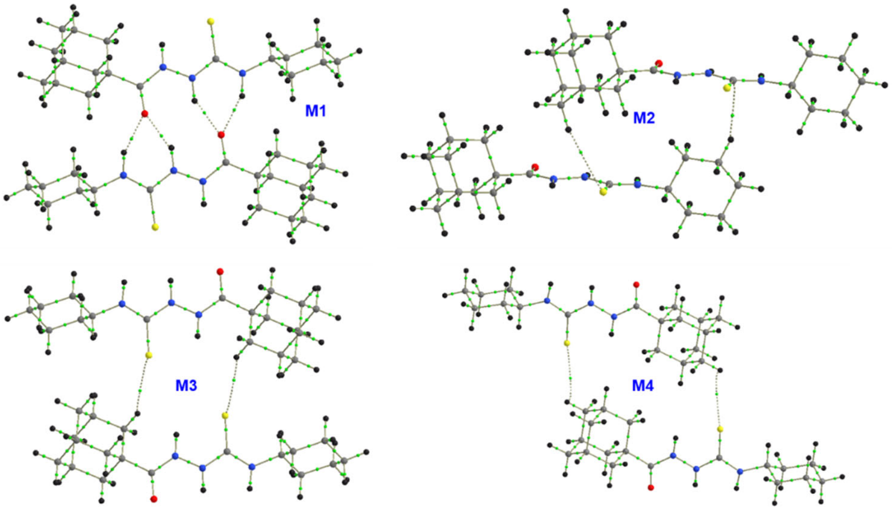

Figure S2. Molecular graphs of various dimers observed in compound **2** showing important intermolecular interactions at their bond critical points.

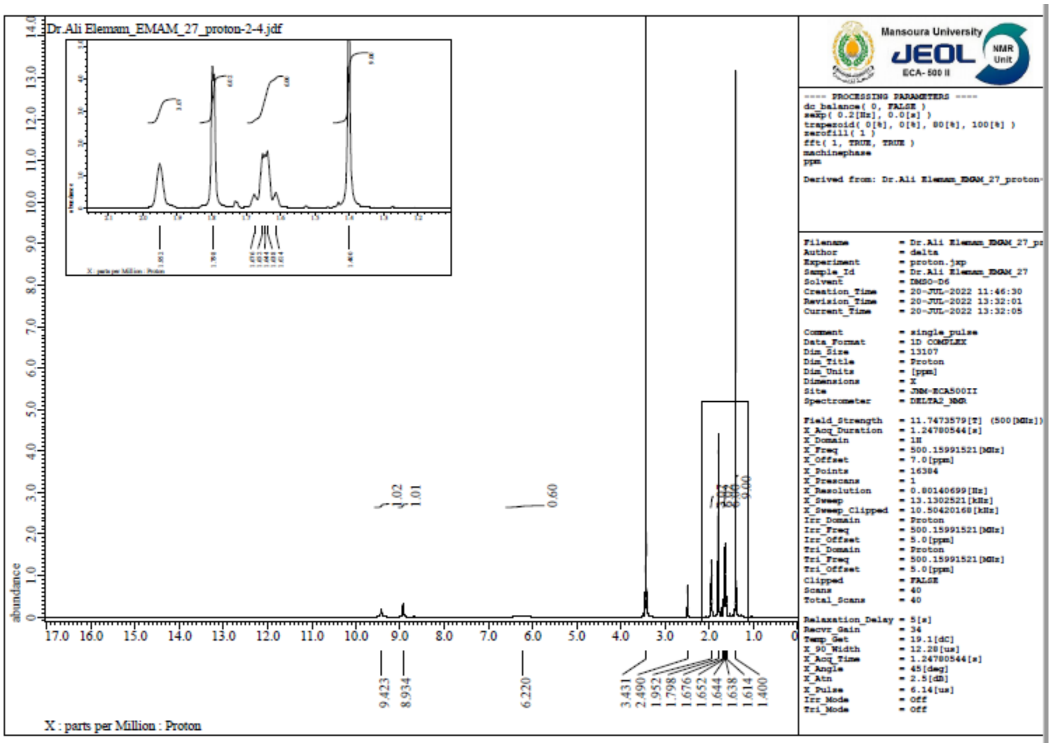

Figure S3. ^{1}H NMR spectrum of compound **1** in DMSO-d_6.

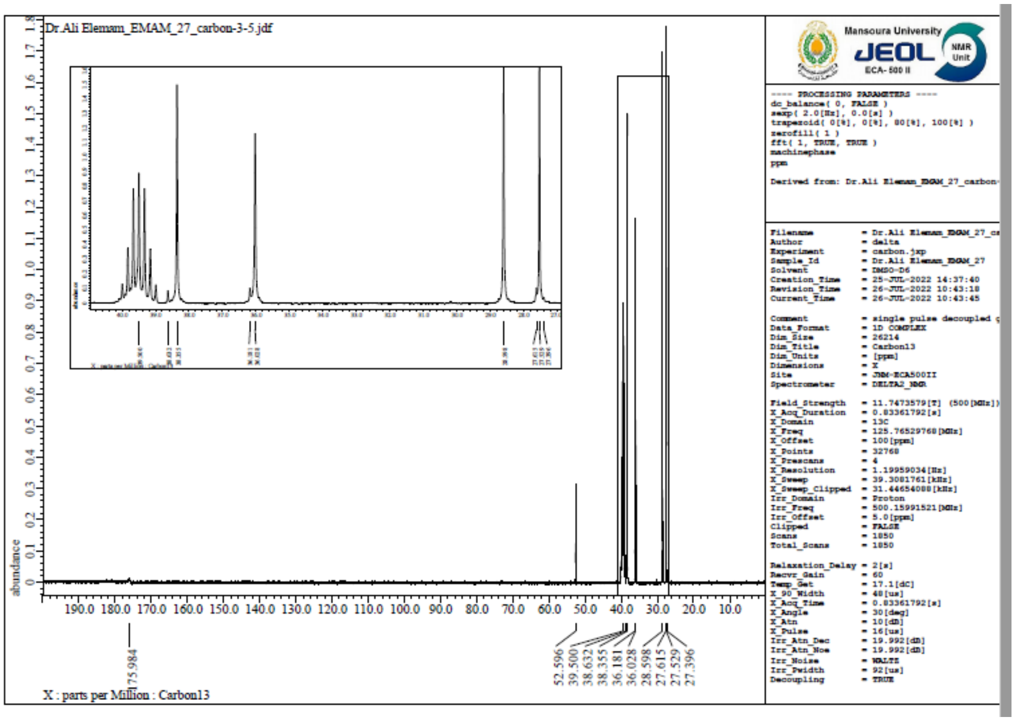

Figure S4. ^{13}C NMR spectrum of compound **1** in DMSO-d_6.

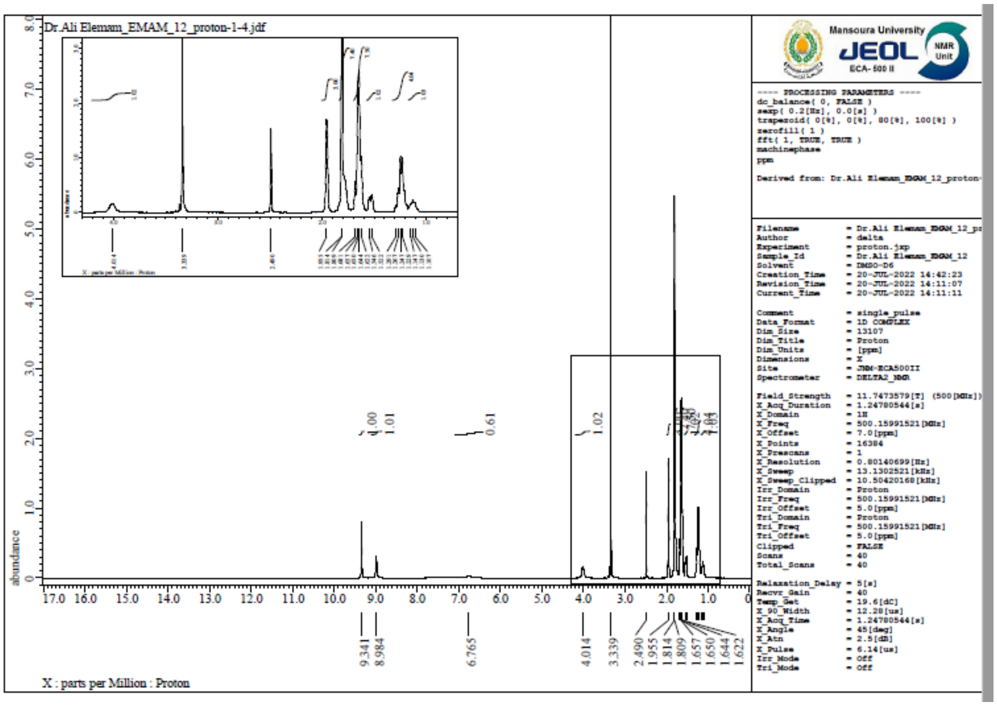

Figure S5. ^{1}H NMR spectrum of compound **2** in DMSO-d_6.

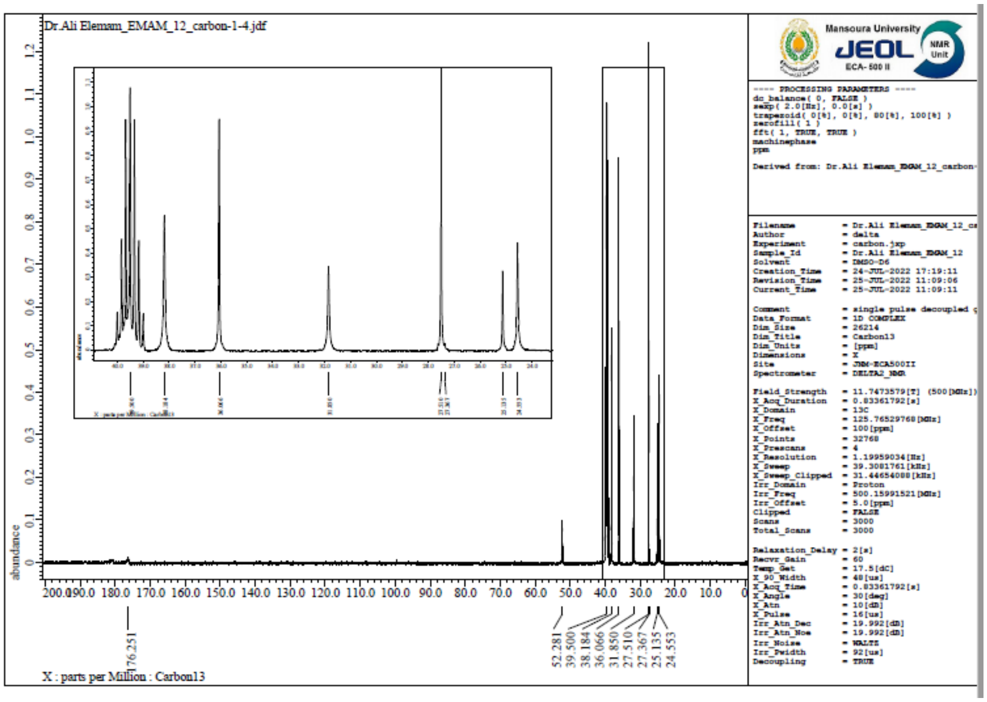

Figure S6. ^{13}C NMR spectrum of compound **2** in DMSO-d_6.

Determination of In vitro Urease Inhibitory Activity

In vitro inhibitory activity of compounds **1** and **2** on Jack bean urease (CAS: 900213-5) was determined using the indophenol method [1]. The concentration of the liberated ammonia was determined using the Berthelot method with phenol-hypochlorite reaction [2], which produces a colored product that is measureable at 630 nm and whose amount is proportionate to urease activity. In 96-well plate reaction mixtures containing phosphate buffer (10 μL of 50 mM K_2HPO_4, pH = 7.0 and 10 μL of double distilled H_2O), Jack

bean urease (1 μg μL^{-1}) was freshly prepared with 50 mM phosphate buffer. Then, 20 μL of the enzyme and 10 μL of different concentrations 0.1–100 μM of compounds **1**, **2** and thiourea in dimethyl sulfoxide were poured in the reaction mixture. The mixture was incubated at 37 °C for 10 min. Enzymatic reactions were started after the addition of 40 μL of the urea substrate and incubation at 37 °C for further 10 min. Urea concentrations (0.156–20 μM) were determined on the basis of urease activity under optimal conditions. Then 80 μL of the alkali reagent (0.5% sodium hydroxide in 0.1% sodium hypochlorite) and 50 μL of 0.005% *w*/*v* sodium nitroprusside, 1.0% *w*/*v* phenol were added to the tested wells and kept at room temperature for 50 min. The amount of liberated ammonia from the reaction mixtures was detected at 630 nm with a microplate reader (BioRad, Washington, USA) and then calculated on the basis of the calibration curves. Inhibition (%) was calculated in accordance with the following equation:

$$\% \text{ Inhibition} = (1 - AT/AC) \times 100 \quad (1)$$

where AT is the absorbance value of the well containing compounds **1**, **2** and thiourea and AC is the absorbance of the reaction mixture without the inhibitors. The IC_{50} values were calculated using nonlinear regression (curve fit) and dose-response inhibition in Graphpad Prism 8.0.2 software (San Diego, CA, USA).

Determination of In vitro Antiproliferative Activity

The in vitro antiproliferative activity screening of compounds **1** and **2** was measured against five human tumor cell lines, namely; PC-3 (human prostate cancer), HCT-116 (human colorectal cancer), HepG-2 (human hepatocellular cancer), HeLa (human cervical cancer) and MCF (human breast cancer) using the 3-[4,5-dimethylthiazoyl-2-yl]-2,5-diphenyltetrazolium bromide (MTT) colorimetric assay [3]. The tumor cells (3000 cells per well) were cultured and seeded into 96-well plates and the plates were incubated for 24 h. The cells were then treated with compounds **1**, **2** and Doxorubicin at different concentrations in DMSO (0.1 μM to 100 μM) at 37 °C in an atmosphere of 5% CO_2 for 48 h. Freshly prepared MTT was added to each well at a terminal concentration of 5 μg/mL and incubated with cells at 37 °C for 4 h. The formazan crystals were dissolved in 100 μL of DMSO in each well, and the absorbency at 492 nm (for absorbance of MTT formazan) and 630 nm (for the reference wavelength) was measured with an enzyme linked immunosorbent assay (ELISA) reader (ChroMate-4300, FL, USA). All compounds were tested three times in each of the cell lines. The IC_{50} values were calculated according to the equation for Boltzmann sigmoidal concentration response curve using the nonlinear regression fitting models (Graph Pad, Prism Version 5). The results reported are means of three separate experiments. Statistical differences were analysed according to one-way ANOVA test wherein the differences were considered to be significant at $p < 0.05$.

References

1. Weatherburn, M. W. Phenol-hypochlorite reaction for determination of ammonia. *Anal. Chem.* **1967**, *39*, 971-974 (DOI: 10.1021/ac60252a045).
2. Tanaka, T.; Kawase, M.; Tani, S. Urease inhibitory activity of simple α,β-unsaturated ketones. *Life Sci.* **2003**, *73*, 2985-2990 (DOI: 10.1016/s0024-3205(03)00708-2).
3. Mosmann, T. Rapid colorimetric assay for cellular growth and survival: Application to proliferation and cytotoxicity assays. *J. Immunol. Methods* **1983**, *65*, 55-63 (DOI: 10.1016/0022-1759(83)90303-4).
